# Hypoattenuating periportal halo on CT in a patient population can occur in presence of a variety of diseases

**DOI:** 10.1371/journal.pone.0260436

**Published:** 2022-01-07

**Authors:** Susann Dressel-Böhm, Henning Richter, Patrick R. Kircher, Francesca Del Chicca

**Affiliations:** 1 Clinic for Diagnostic Imaging, Vetsuisse Faculty, University of Zurich, Zurich, Switzerland; 2 Vetimage Diagnostik GmbH, Oberentfelden, Switzerland; Dicle University: Dicle Universitesi, TURKEY

## Abstract

Many pathologies can occur in the periportal space and manifest as fluid accumulation, visible in Computed tomography (CT) images as a circumferential region of low attenuation around the intrahepatic portal vessels, called periportal halo (PPH). This finding is associated with different types of hepatic and extra-hepatic disease in humans and remains a non-specific sign of unknown significance in veterinary literature. The aim of this study was to investigate the prevalence of PPH in a population of patients undergoing CT examination and to assess the presence of lesions related to hepatic and extra-hepatic disease in presence of PPH. CT studies including the cranial abdomen of dogs and cats performed over a 5-year period were retrospectively reviewed. The prevalence of PPH was 15% in dogs and 1% in cats. 143 animals were included and the halo was classified as mild, moderate and severe, respectively in 51%, 34% and 15% of animals. The halo distribution was generalized in 79 cases, localized along the second generation of portal branches in 63, and along the first generation only in one. Hepatic disease was present in 58/143 and extra-hepatic disease in 110/143 of the cases. Main cause of hepatic (36%) and extra-hepatic disease (68%) was neoplasia. Associations between halo grades and neoplasia revealed to be not statistically significant (p = 0.057). In 7% of animals the CT examination was otherwise unremarkable. PPH is a non-specific finding, occurring in presence of a variety of diseases in the examined patient population.

## Introduction

The periportal space is an anatomic region that surrounds the portal vein (PV) and its branches and is embedded in a loose connective tissue that also contains the hepatic artery, bile duct, nerves and lymphatic vessels. Peritoneal fat is frequently seen around the portal vein at the level of the porta hepatis as a normal finding [1]. The PV and its collaterals are highly attenuating in the computed tomography (CT) post-contrast venous phase and surrounded by homogenous hepatic parenchyma [2]. A variety of pathologies can occur in the periportal space via hematogenous, biliary, lymphatic, neural and peritoneal routes and manifest as fluid accumulation. This leads to a distention of the interstitial tissue around the portal triad structures and appears as a circumferential region of low attenuation parallel to the intrahepatic portal system, called periportal halo (PPH) [1]. These circumferential regions parallel to the portal branches are hyperattenuating (attenuation range -3 to 10 Hounsfield units) [3] compared to the density of fat tissue [4]. Other synonyms that have been used in the radiology literature include “periportal collar”, “periportal tracking”, “periportal ring” or “tram line” and “periportal hypodense zones” [5–7].

This CT finding has been described in different hepatic and extra-hepatic diseases in humans and reflects altered hepatic lymphatic dynamics due to fluid overload or lymphatic obstruction or congestion. In a descriptive study, lymph stasis after interruption of the lymphatic vessels during liver transplantation was observed post-operatively in 3 human patients and was simulated in piglets revealing numerous dilated lymph vessels and lymphedema on histopathology [3].

Causes of periportal edema in people are numerous and include hepatic and extra-hepatic disorders such as acute hepatitis [1, 8], veno-occlusive disease after liver and bone marrow transplantation [1, 9], overhydration [10] or trauma-related pathologies [5, 11, 12]. The latter is leading to hypovolemic shock and subsequent centralization, intrabdominal hematoma formation and can induce lymph edema or distention of lymphatics by rapid intravenous fluid administration during resuscitation. Other causes such as acute pyelonephritis with concomitant severe systemic sepsis [13, 14] and cardiac disease have also been reported. In particular, it has been described in congestive heart failure with secondary hepatic congestion [1, 5], cardiac tamponade [15] and pericarditis in children [16]. Furthermore, space-occupying lesions in the region of the porta hepatis or hepatic lymphadenomegaly [5] accompanied with neoplasia or inflammatory benign lesions might occur in presence of PPH [1].

In veterinary literature, the presence of a PPH has been reported only as a non-specific CT feature in presence of portosystemic shunts [17]. In clinical practice PPH is occasionally seen, but literature about the possible clinical significance and prevalence is substantially lacking.

The goals of the study were as follows: (a) investigate the prevalence of PPH in a large patient population of dogs and cats, (b) assess the presence of other concomitant tomographic findings related to hepatic or extra-hepatic disease, (c) test a possible association between PPH presence or grade and hepatic or extra-hepatic disease.

## Materials and methods

This study was conducted in a retrospective, bi-institutional and descriptive design to evaluate the presence of PPH based on abdominal CT examinations of dogs and cats presented to the Vetsuisse Faculty, University of Zurich, Switzerland (Institution 1) and Vetimage Diagnostik GmbH (Institution 2) for various purposes between January 2016 and December 2020. No additional stress or pain for animals was caused by this retrospective data analysis. All data was obtained from patients during clinical routine work-up. The retrospective use of imaging data does not require animal permission according to Swiss Animal Welfare Act.

Written owner’s consent was obtained for each patient prior to diagnostic work-up to use data for research purposes.

Final decisions for subject inclusion or exclusion were made based on jointly consensus of 2 of the authors (SD and FDC), taking into account the following objective criteria:

Animals were included in the study if a PPH was present in a complete CT examination (complete meaning consisting of pre- and late post-contrast series, imaging the entire liver, the PV and its tributaries) and if medical records of the patients were available for review (including signalment, anamnestic, clinical information, biochemical analysis as well as results of cytological or histological examination if performed). In the included animals the biochemical analysis, when available had to be performed within 7 days prior to the CT examination.

Animals were excluded from the study if no PPH was present. Cases where the PPH was present, but the CT examinations were incomplete or no medical data were available for review were also excluded.

Helical CT studies of the abdomen were acquired using 16-slice CT scanners (Institution 1: Brilliance CT, Philips, Zurich, Switzerland; Institution 2: GE Lightspeed, Optima CT520, GE Healthcare, Glattbrugg, Switzerland), most animals being positioned in ventral, occasionally in dorsal recumbency. All patients underwent general anesthesia. Respiratory apnea was induced during scanning. Standard CT examination protocols were followed in each institution. Scanning parameters performed with the Philips Brilliance 16 included: Slice thickness 1–1.5 mm, 120kVp, 183–229 mA, collimator pitch 0.688. Scanning parameters performed with the GE Optima CT520 included: slice thickness 0.625–1.25 mm, 120 kVp, 235 mA, and collimator pitch 0.562. For contrast-enhanced studies, all patients received 2ml/ kg iodinated non-ionic contrast medium (Accupaque 350, 350 mg of I/ml, GE Healthcare, Glattbrugg, Switzerland). The bolus was injected using either a programmable injector Accutron CT-D Medtron Injector (SMD Medial AG, Trägerwilen, Switzerland) with an injection rate of 2 ml/s (Institution 1) or manually (Institution 2). At least one post-contrast CT series was performed in every patient in a delayed venous phase (90–120 seconds after starting the injection of contrast medium).

CT studies were retrieved from the Picture Archiving and Communication System (PACS) and transferred to a workstation with a vendor-specific post-processing software: Philips IntelliSpace PACS Radiology (Philips Medical Systems, Best, Netherland at Institution 1) and Horos^TM^ (Horosproject.org, Annapolis, USA at Institution 2). Images were reviewed in a dynamically adjustable soft tissue window setting (window width/ level: 400/40) using transverse and multiplanar reconstructions for interpretation.

**Fig 1:**
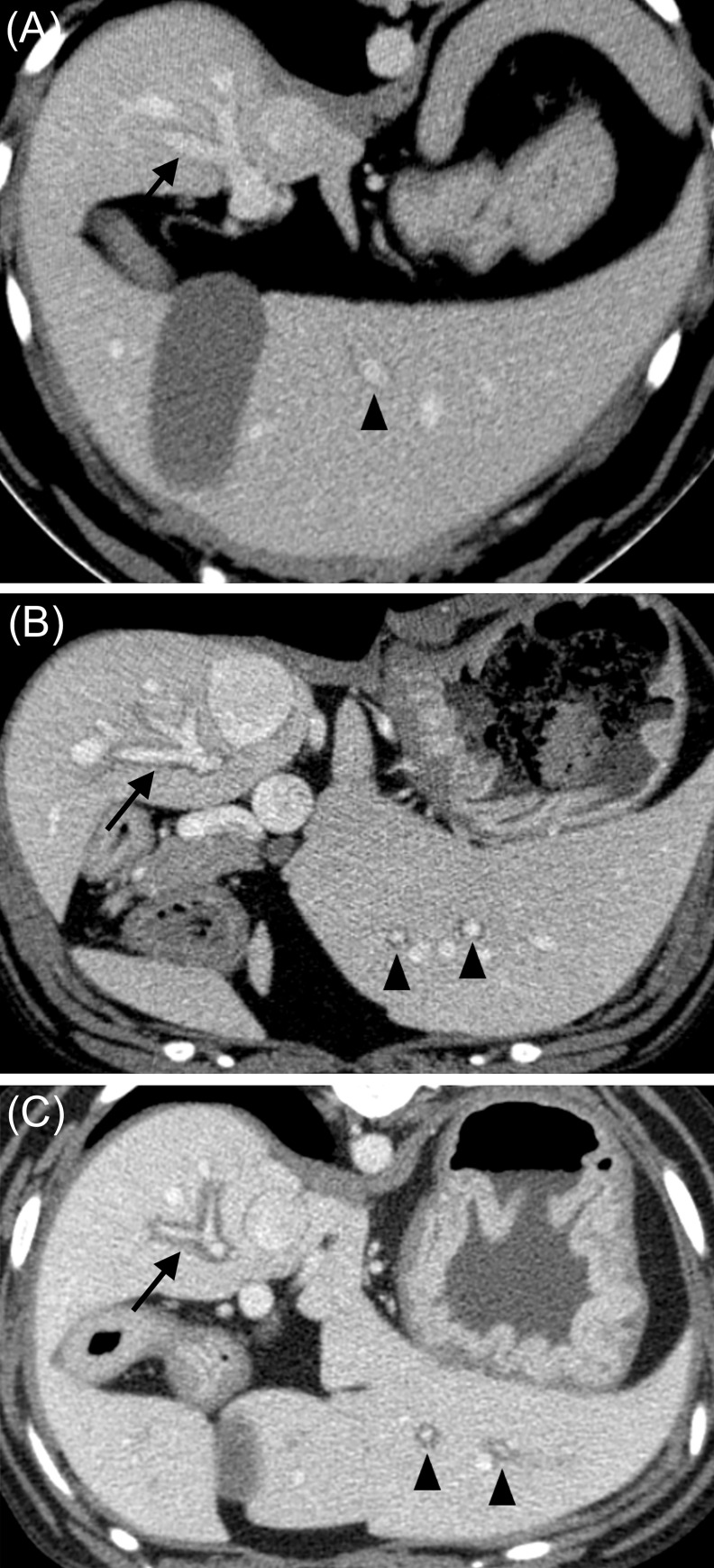
Helical contrast-enhanced axial CT images of the intrahepatic PV of 3 different periportal halo grades. (A) halo grade mild, (B) halo grade moderate, and (C) halo grade severe. Note the more horizontal oriented right branch and the right lateral lobe branches creating the appearance of “tram lines” (arrow). The segmental branches of the depicted left liver lobes are more vertically oriented and therefore creating “rings” or “collars” (arrow heads). Abbreviations: CT, Computed Tomography; PV, portal vein.

The following CT findings were evaluated for each patient included in the study: Grade of the PPH, evaluated subjectively according to its thickness. Patients were allocated into 3 groups based on the assigned grade—mild, when the halo was hairline thin, moderate and severe, with progressively increasing thickness of the PPH. When classified as severe, the PPH had the similar thickness as the diameter of the corresponding portal vessel, as shown in Fig 1. Grading was performed by an ECVDI-certified veterinary radiologist (FDC) and an ECVDI resident (SD) based on jointly consensus without knowledge of the patient’s signalment or history at the time of recording.

**Fig 2:**
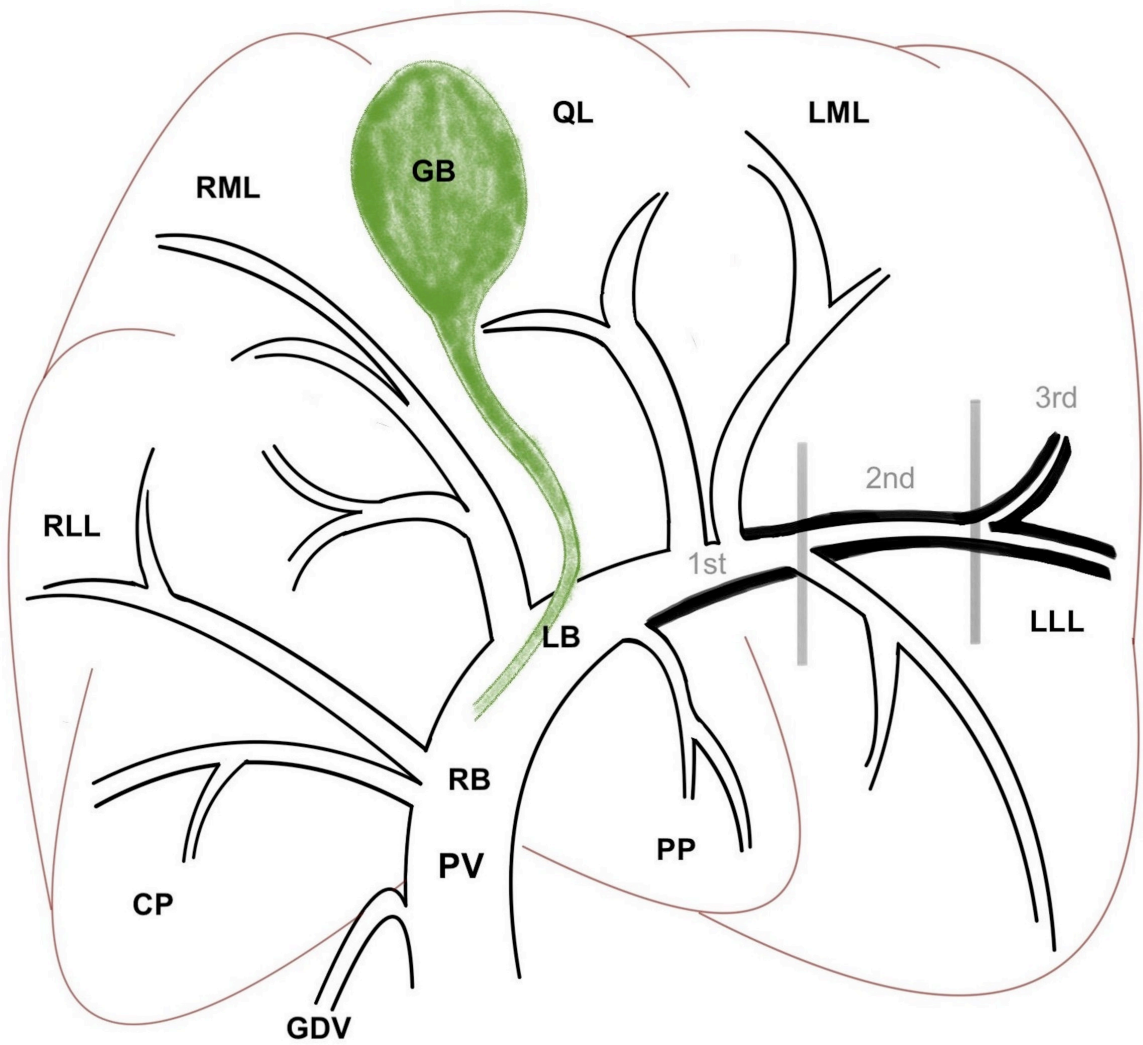
Schematic drawing of the intrahepatic portal system. Dorsal plane image showing the short right (RB) and long left branch (LB) which are further dividing into lobe and segmental branches. An example of the periportal halo is given along the left lateral lobe branch and is presented as thickened black line. Classification of the halo distribution into first (1^st^), second (2^nd^) and third (3^rd^) generation is marked with vertical lines. Abbreviations: PV, portal vein; GDV, gastroduodenal vein; CP, caudate process of caudate lobe; RLL, right lateral lobe; RML, right medial lobe; QL, quadrate lobe; LML, left medial lobe; LLL, left lateral lobe; PP, papillary process of caudate lobe; GB, gall bladder.

Distribution of the halo was assessed depending on the involvement of the complete first, second or third generation of portal branches based on the ramification of the intrahepatic PV as described in the literature [18] (see Fig 2). The PV system with its tributaries and intrahepatic branches were evaluated based on its shape, subjective size, position, presence of filling defects or aberrant vessels.

Liver morphology was assessed subjectively according to its size (normal, hepatomegaly, microhepatia) and parenchymal appearance (homogenous, heterogenous). The liver parenchyma was termed “homogenous” when all lobes showed a uniform attenuation in pre- and post-contrast series. It was defined as “heterogenous” when the parenchyma revealed areas of altered attenuation and enhancement pattern compared to normal liver parenchyma. Intrahepatic lesions, if present, were described as unstructured/patchy, nodular (< 3cm) or mass (≥ 3cm) [19]. Hepatic lymph node size was evaluated on the basis of published normal CT lymph node characteristics [20]. Enlargement was subjectively classified as mild, moderate or severe. The presence of peritoneal and/or retroperitoneal effusion was noted.

Furthermore, studies and medical records were evaluated for signs of acquired portal collateral circulation [21] (including splenogonadal shunts and varices), trauma, chronic heart failure (CHF) and pyelonephritis. These specific pathologies were investigated based on the literature on human medicine. Liver enzyme values of alanine aminotransferase (ALT) and alkaline phosphatase (AP) were reviewed for detection of biochemical abnormalities. These specific liver enzymes were investigated, as routinely assessed in our institutions when hepatopathy is suspected. Every patient was assigned to a category of final diagnosis: hepatic disease, extra-hepatic disease, combination of hepatic and extra-hepatic disease or no final diagnosis. The final diagnosis ‘confirmed’ hepatic or extra-hepatic disease was made based on the cytological and/or histopathological evaluation of the respective organ and/or pathognomonic CT findings (e.g. in the case of portosystemic shunt). If a definitive diagnosis could not be confirmed cytohistologically, the animals were classified as ‘suspected’ hepatic or extra-hepatic disease. In case of multiple organ system involvement, patients were classified as concurrent hepatic and extra-hepatic disease. Consensus was reached between the first and last author to determine the final category for each animal based on review and discussion of all available anamnestic and clinical information, laboratory and imaging findings. Patients without a conclusive diagnosis were classified as ‘no final diagnosis’.

### Statistical analysis

Statistical analyses were performed by 2 of the authors, a diagnostic imaging resident (SD), supervised by a veterinarian with a certificate of advanced studies (CAS) in applied statistics (HR). Data were coded in Excel version 16.45 (Microsoft®, Redmond, USA) and analyzed with SPSS version 27.0 (IBM SPSS Inc, Armonk, USA). Statistical analysis was provided for the whole set of data, as well as for species-specific subsets. Descriptive statistics was performed, including mean/median and standard deviation/range. For discrete variables relative frequencies were calculated.

Non-parametric tests were applied between multiple (Kruskal Wallis) or two independent variables (Wilcoxon test). Association between ordinal-scaled variables was tested with Spearman-Rho test. Overall, p-values < 0.05 were considered to be statistically significant.

## Results

In total, 1168 abdominal CT examinations were reviewed for Institution 1, and 125 of 858 (15%) dogs and 4 of 310 (1%) cats showed PPH on CT studies. A second group of patients from Institution 2 were included through medical re-call.

In total, 143 animals met the inclusion criteria in the selected period of time. There were 138 canine and 5 feline patients. Most of the dogs were mixed-breed (n = 33), followed by Labrador Retriever (n = 19), Golden Retriever (n = 9), German Boxer (n = 6), German Shepherd (n = 5), French bulldog (n = 5), Rottweiler (n = 3), Flat Coated Retriever (n = 3), Malinois (n = 3) and a variety of other breeds that were presented once or twice. Included cats were: 2 domestic shorthair, 1 each British shorthair and Maine Coon, and one of unknown breed.

The overall age of the animals ranged from 0.3 to 16.8 years (median 8.4 years). There were 75 males (38 intact and 37 castrated) and 68 females (13 intact and 55 spayed).

In 73 (51%) of the included animals the PPH was classified as mild, in 49 (34.3%) as moderate and in 21 (14.7%) as severe.

The distribution of the halo was generalized in 79 of 143 (55.2%) cases, localized along the first and second generation of intrahepatic portal branches only in 63 of 143 (44.1%), and along the first generation only in 1 of 143 (0.7%). The halo seen along the first generation only was graded as mild. Periportal halo distributed along the first and second generation was graded as mild in 48 animals, moderate in 13, and severe in 2. Generalized halo was graded as mild in 24 animals, moderate in 36, and severe in 19. In 26 of 143 cases a similar, hypoattenuating halo was additionally noted around the hepatic veins.

56 of 143 (39.2%) patients had morphological (size and/or parenchymal) and/or vascular hepatic abnormalities. 16 of 143 (11.2%) patients had generalized and 17 of 143 (11.9%) focal hepatomegaly, 2 patients (1.4%) showed microhepatia, and in the remaining 108 (75.5%) cases, the liver size was considered normal. The liver parenchyma was homogenous in 99 of 143 (69.2%) of the cases; the parenchyma was nodular in 20 of 143 (14%), unstructured patchy in 9 (6.3%), and in 15 of 143 (10.5%) patients hepatic mass/es were present. Aberrant vessels were seen in 6 of 143 (4.2%) animals: in 4 cases multiple shunting vessels were apparent, 2 of them with concurrent filling defects within the PV, and in 2 cases the shunt was single. Liver morphology did not show significant difference between halo grades (dogs: p = 0.298; cats: p = 0.800).

Hepatic lymphadenomegaly was present in 52 of 143 (37.4%) patients, of which the majority was graded as mild (40 of 52). Moderate lymphadenomegaly was diagnosed in 10 (7%), and severe only in 2 (1.4%) dogs. Peritoneal and retroperitoneal effusion was identified in 35 (24.5%) and 4 animals (2.8%), respectively. 3 animals had a history of trauma and were involved in a road traffic accident. Signs of pyelonephritis and CHF were present in 2 and 4 patients, respectively. All patients with CHF were dogs and showed varies cardiac pathologies: 2 patients with large heart base tumors developed signs of congestive hepatomegaly due to compression of the caudal vena cava and the hepatic veins, 1 patient developed pulmonary hypertension due to pressure overload and the last dog had mild pulmonary edema resulting from bilateral severe auricular and atrial dilatation.

Splenogonadal shunts and esophageal varices were identified in none of the patients.

Primary hepatic disease was present in 58 (40.6%) out of 143 patients, of which 28 were confirmed by histology or cytology, the remaining cases diagnosed through imaging diagnosis and medical record review. In 3 cases the hepatopathy was suspected as the primary cause of disease, and in 27 was suspected secondary or concurrent to a primary extra-hepatic disease. The majority of the patients (exclusively dogs) with hepatic disease had a neoplasia (21 out of 58, 36.2%) based on cytological or histopathological analysis. 2 of the cytologically confirmed hepatic mast cell tumors revealed no morphological abnormalities on CT examinations other than PPH.

There was no statistically significant difference between the occurrence of hepatic neoplasia and halo grade (p = 0.057).

Most animals had an extra-hepatic disease (110 out of 143, 76.9%). 89 cases were confirmed by cytology and/or histopathology and 21 cases were suspected. The main cause of extra-hepatic disease (75 of 110, 68.2%) was neoplasia. No significant difference between extra-hepatic disease and halo grade was detectable (dogs: p = 0.914, cats: p = 0.800). Associations between halo grades and neoplasia (hepatic or extra-hepatic origin) was not statistically significant (dog: n = 128, spearman: -0.138, p = 0.121; cats: n = 5, spearman: -0.152, p = 0.807).

Liver biochemical tests were available in 108 of 143 animals and were performed on the same day as the CT examination in 77 patients, within 24 hours in 13 patients and between 2 and 7 days prior to the CT examination in 18 patients. 54 patients had normal AP and ALT levels. Liver enzyme abnormalities were noted in 54 animals (50%), 23 of which had increased AP and ALT levels, and 31 showed elevations in only AP or ALT. Biochemical abnormalities and halo grades did not differ significantly (dogs: p = 0.162; cats: p = 0.709; total p = 0.110). Table 1 summarizes patient data by hepatic, extra-hepatic, concurrent hepatic and extra-hepatic disease and patients with no final diagnosis.

**Table 1:** Summary of patient data sorted by hepatic, concurrent hepatic + extra-hepatic, extra-hepatic disease and no final diagnosis.

Total (n = 143)*	Hepatic disease (n = 58)		Concurrent Hepatic + Extra-hepatic disease (n = 35)		Extra-hepatic disease (n = 75)		No final diagnosis (n = 10)	
**Category of pathologies**	Neoplasia	21	Neoplasia	25	Neoplasia	50		
	Hepatitis	2	Inflammation	5	Inflammation	21		
	PSS (congenital)	3	Congenital anomaly	1	Congenital anomaly	2		
	PSS (acquired)	3	Degeneration	1	Degeneration	1		
	Congestion	5	Hematoma	1	Endocrine	1		
	Steroid hepatopathy	3	Unknown etiology	2				
	Abscess	1						
	Lipidosis	1						
	Granuloma	1						
	Hyperplasia	1						
	Cyst	3						
	Unknown etiology	15						
**Liver biochemical tests (n = 108)**	AP+ALT elevated	17	AP+ALT elevated	6	AP+ALT elevated	6	AP+ALT elevated	0
	AP or ALT elevated	15	AP or ALT elevated	10	AP or ALT elevated	15	AP or ALT elevated	1
	Normal biochemistry	13	Normal biochemistry	9	Normal biochemistry	36	Normal biochemistry	5
	n.a.	13	n.a.	10	n.a.	18	n.a.	4
**Hepatic morphological and/or vascular abnormalities (n = 56)**	Abnormal	56	Abnormal	33	Abnormal	0	Abnormal	0
	Normal	2	normal	2	Normal	75	Normal	10
**Hepatic lymphadenomegaly (n = 52)**	Mild	19	Mild	9	Mild	20	Mild	1
	Moderate	6	Moderate	3	Moderate	4	Moderate	0
	Severe	2	Severe	1	Severe	0	Severe	0
	Normal hepatic LN	31	Normal hepatic LN	22	Normal hepatic LN	51	Normal hepatic LN	9

Abbreviations: PSS, portosystemic shunt; AP, alkaline phosphatase; ALT, alanine-aminotransferase; LN, lymph nodes; n.a., not assessed.

*Please note: total patient number is composed of the number of patients categorized as hepatic disease, extra-hepatic disease and no final diagnosis. Patients with both hepatic and extra-hepatic disease were primarily counted in the hepatic or extra-hepatic category. This category was determined depending on the most likely clinically relevant pathology at the time of imaging, i.e., hepatic or extra-hepatic pathology responsible for the symptoms at the time of imaging.

In 10 animals (7%) the only CT finding was the PPH and the examination was, except for one patient with mild hepatic lymphadenomegaly, unremarkable. Liver cytology was available in 4 and histology in 1 of these cases and was considered normal. Most of these patients were presented for staging or re-staging of mast cell tumors that developed (sub)cutaneously or at mucocutaneous junctions.

## Discussion

Periportal edema manifests on CT as hypoattenuating zones paralleling the intrahepatic portal branches and is associated with a variety of hepatic and extra-hepatic pathologies that are poorly described in veterinary medicine. While etiologies described in humans are multifold, the PPH has only been mentioned in presence of portosystemic shunts in dogs and cats [17]. In this study, we describe the prevalence of PPH in a subpopulation of canine and feline patients (retrospective study with selection bias) and categorize the diseases where a PPH is present.

PPH was more prevalent in dogs (15%) than in cats (1%). The occurrence of the PPH is more frequent in both species compared to humans, in which the halo was detected in 20 of 2500 cases over an 8-month period [5]. The reason for different prevalence among species remains speculative. Livers from cats whose thoracic ducts were obliterated showed a dilatation of the portal lymphatic vessels and expansion of the sublobular interstitial space with an increased infiltration of mast and plasma cells [22]. The release of connective tissue mediators promoted by mast and plasma cells involves angiogenetic and fibrogenetic processes to reduce hepatic edema caused by induced lymphstasis. This could be a possible reason for the described lower prevalence in cats compared to dogs. It still remains unknown whether this pronounced cell proliferation is species-specific.

The hepatic lymph drainage in dogs and cats is divided into a superficial and deep system. The superficial system (hilar) runs in the areolar zone along the PV and supplies approximately 80% of the hepatic lymph drainage. The remaining 20% follows the hepatic veins (hepatic venous lymph system) and can be drained by superficial lymphatics via anastomoses [23]. In 26 out of 143 cases, a concomitant halo was detected around the hepatic veins and might be explained by the direct relation of hepatic lymph flow to hepatic venous pressure in dogs and cats [24]. This explanation could be supported by the fact that patients with known pressure overload (right-sided cardiac failure and 1 case with Budd-Chiari syndrome) are found among patients with halo around the portal and the hepatic vessels. On the other hand, this known pressure overload was present in only 2 out of 26 patients with concomitant halo around the hepatic veins and it is likely that other physiologic or pathophysiologic mechanisms are involved in the presence of perivascular halo. Another possibility is that pressure overload may have been present with no detected secondary signs on CT and not recorded in the medical records.

The study population consists of a heterogenous group of dogs and cats with various diseases reflecting the incidence of different pathologies at our institutions. The majority of the patients was referred to the oncology department of Institution 1 for initial diagnosis, staging, surgical and radiation treatment planning, which explains the high number of oncologic patients, with both hepatic or extra hepatic neoplasia. As the patients of our population are middle to older age, the likelihood that they suffer age-related disease is high. Both facts represent an unavoidable bias and may explain the overrepresentation of patients with neoplastic etiology in this study. However, in the studied patient population no association was present between presence or degree of PPH and a neoplastic process.

Presence of PPH was also not associated to a specific breed, gender or gonadal status of the analyzed population.

Interestingly, none of the cats with PPH had a confirmed hepatic neoplasia.

**Fig 3:**
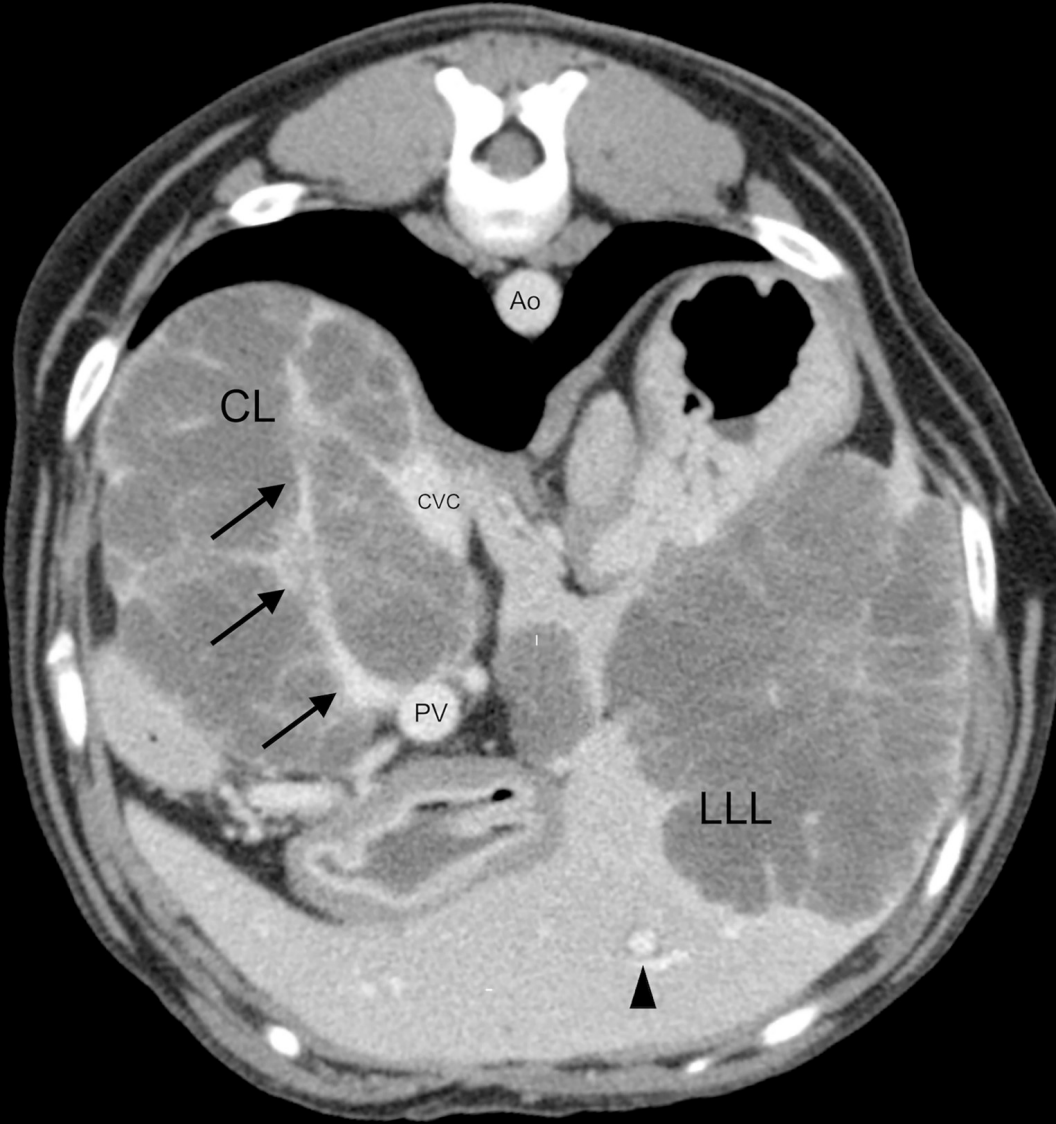
Helical contrast-enhanced CT image of a dog with a cholangiocarcinoma. Axial CT image showing multiple lobulated non-enhancing hypoattenuating masses in the caudate and left lateral lobe of the liver which were histopathologically confirmed as cholangiocarcinoma. Note how the tumor encases the intrahepatic portal branch and reduces the vessel’s diameter (arrows). The CVC is severely flattened and compressed by the mass effect of the tumor. There is a concomitant mild periportal halo seen in the periphery of the left lateral lobe (arrow head). Abbreviations: CT, Computed Tomography; PV, portal vein; CVC, caudal vena cava, Ao, Aorta; CL, caudate lobe; LLL, left lateral lobe.

The majority of the cases suffered neoplastic processes in presence of PPH, but it is imperative to differentiate edema like lesions resulting from dilated obstructed lymphatics from periportal neoplastic infiltrate. The latter is creating a mass effect with periportal hypoattenuating regions usually segmentally or focally distributed with asymmetrically thickened halos [25, 26]. Even in known malignancies, PPHs can result from non-neoplastic causes like periportal edema [26]. Fig 3 shows a dog with confirmed hepatic neoplasia (cholangiocarcinoma) infiltrating several lobes and surrounding the intrahepatic portal branch of the caudate lobe. Generalized mild PPH and halo around the hepatic veins was concurrently seen along all the intrahepatic portal and hepatic venous branches, respectively, also not directly adjacent to the visible neoplasia. This neoplasia caused severe compression and intra-operatively confirmed adhesions to the CVC, and mild compression of the extra-hepatic PV (not shown). Edema not adjacent to the neoplasia can have been caused in this case by the severe vascular compression leading to secondary obstructed lymphatic drainage.

In only 34.7% of the patients lymphadenomegaly of the hepatic lymph nodes was present. In most of these patients lymphadenomegaly was graded as mild or moderate, with only 2 dogs having severe lymphadenomegaly. For this reason, it is highly unlikely that the PPH is directly correlated to compression of the PV secondary to enlarged lymph nodes.

Only a minority of the animals included in the present study (n = 6) had a vascular abnormality consisting with shunt/s, confirming that, also in veterinary medicine, the PPH is not a specific sign and possible etiologies go far beyond this single reported pathology so far.

The retrospective and descriptive nature of the study is a clear limitation of this study, which comes along with selection bias for the retrospective available subpopulation and does not allow independent randomization. Additionally, the retrospective approach of the study prevented histopathological evaluation of each patient’s liver with specific attention to the vascular structures and the periportal space to confirm diagnosis of periportal edema, which is considered one of the main limitations of the current study. One of the included dogs with histopathologically confirmed PV hypoplasia was found to have portal fibrosis and moderate dilatation of the periportal lymphatics, which is in accordance with former reports [5]. Based on the limited number of available histopathologically confirmed cases, we therefore refer to the PPH as periportal edema like lesions.

Blunt trauma is considered to cause PPH due to the presence of blood in the periportal space in human medicine. Only 3 patients (2 dogs and 1 cat) of our population had a history of previous trauma and the CT scan was performed with this indication. None of these 3 patients showed trauma related injuries of the liver on CT suggesting that in these cases the occurrence of PPH may not be directly related to hepatic injury. A described possible explanation for the presence of PPH can be attributed to a rapid expansion of the intravascular volume during intravenous fluid administration in course of patient stabilization [11]. In our study, we can only speculate about the role of the intravenous fluid administration on the presence of PPH, because all animals were clinical patients and anesthetic protocols were selected on a case-by-case basis by clinicians of the anesthesiology service. In particular, details about the drugs and intravenous fluids administered over time are not known. Variable factors regarding intravenous fluid administration (like amount and kind of fluid, rate of fluid administration over time, the hydration status of the patient) could play a role for the presence of PPH, also considering that every patient receives an intravenous catheter and fluid administration before CT examination for stabilization, anesthetic procedure and contrast injection.

Similarly, for our animal population, presence of PPH is not specific for a hepatic disease as seen in human literature. In fact, more than half of the patients with PPH were diagnosed with an extra-hepatic disease.

Follow-up information to monitor persistence, progression or resolution of the PPH over time were not available in our patients and was beyond the scope of this study. It would be interesting to investigate if this tomographic sign reflects a particular temporary condition of the patient or if it persists until resolution or regression of the concurrent disease occurs.

Furthermore, there was no attempt to correlate the PPH to neither the severity nor the duration of biochemical abnormalities. Of 54 patients with elevation of AP, ALAT or both enzymes, in 22 cases no underlying hepatic pathology was diagnosed and the origin of the biochemical abnormalities remains unknown. Moreover, the recorded biochemical abnormalities were present at the time of the CT examination, and likely reflect the functional status of the liver at the time of imaging, but previous presence or persistence of these abnormalities have not been investigated.

In a small number of patients (n = 10) no abnormalities were detected on the CT examination other than the presence of PPH. This fact raises the question if PPH could be an incidental finding with no clinical significance. That is of course very difficult to prove, considering that no healthy control group of animal undergoes a CT examination.

The selection of the patients was based on the CT routine protocol examination established at our institutions in the last 5 years. The lack of image acquisition during the arterial and portal phase [27] could have let some hepatic lesions unrecognized and possible presence of PPH only in the portal phase undetected.

Lastly, a small number of cats was included. The lower prevalence of the PPH in cats compared to dogs prevents an overall statistical analysis including all the animals.

## Conclusion

CT visible PPH is a non-specific finding in dogs and cats. PPH occurred in the analyzed patient population in presence of a variety of diseases, hepatic and extra-hepatic, of neoplastic and non-neoplastic origin. The study proves that the presence of PPH is not limited to the few pathologies reported so far. Future work should focus on the hydration status of the patient, the amount of fluid administration, possible reversibility of the finding and focused histopathological examination.
